# Experimental Evidence for Phonemic Contrasts in a Nonhuman Vocal System

**DOI:** 10.1371/journal.pbio.1002171

**Published:** 2015-06-29

**Authors:** Sabrina Engesser, Jodie M. S. Crane, James L. Savage, Andrew F. Russell, Simon W. Townsend

**Affiliations:** 1 Institute of Evolutionary Biology and Environmental Studies, University of Zurich, Zurich, Switzerland; 2 Department of Animal & Plant Sciences, University of Sheffield, Sheffield, United Kingdom; 3 Department of Zoology, University of Cambridge, Cambridge, United Kingdom; 4 Centre for Ecology & Conservation, College of Life & Environmental Sciences, University of Exeter, Penryn, Cornwall, United Kingdom; 5 Fowlers Gap Arid Zone Research Station, School of Biological, Earth & Environmental Sciences, University of New South Wales, Sydney, Australia; Princeton University, UNITED STATES

## Abstract

The ability to generate new meaning by rearranging combinations of meaningless sounds is a fundamental component of language. Although animal vocalizations often comprise combinations of meaningless acoustic elements, evidence that rearranging such combinations generates functionally distinct meaning is lacking. Here, we provide evidence for this basic ability in calls of the chestnut-crowned babbler (*Pomatostomus ruficeps*), a highly cooperative bird of the Australian arid zone. Using acoustic analyses, natural observations, and a series of controlled playback experiments, we demonstrate that this species uses the same acoustic elements (*A* and *B*) in different arrangements (*AB* or *BAB*) to create two functionally distinct vocalizations. Specifically, the addition or omission of a contextually meaningless acoustic element at a single position generates a phoneme-like contrast that is sufficient to distinguish the meaning between the two calls. Our results indicate that the capacity to rearrange meaningless sounds in order to create new signals occurs outside of humans. We suggest that phonemic contrasts represent a rudimentary form of phoneme structure and a potential early step towards the generative phonemic system of human language.

## Abstract 2

The chestnut-crowned babbler, a social species of bird from the Australian outback, possesses the basic capacity to generate words, shedding new light on the origins of word generation in our own species.

## Author Summary

A major question in language evolution is how its generative power emerged. This power, which allows the communication of limitless thoughts and ideas, is a result of the combinatorial nature of human language: meaningless phonemes can be combined to form meaningful words (*phonology*), and words can be combined to form higher-order, meaningful structures (*syntax*). While previous work has indicated the potential for animals to form syntax-like constructions, there exists little convincing evidence for a basic phonemic capacity in animals. Here, we demonstrate, using analyses combined with natural observations and playback experiments, that the cooperatively breeding chestnut-crowned babbler reuses two meaningless acoustic elements to create two functionally distinct vocalizations. This result suggests the basic ability for phoneme structuring occurs outside of humans and provides insights into potential early evolutionary steps preceding the generative phonemic system of human language.

## Introduction

The vast lexicons that characterise human languages are the product of physical and cognitive processes that guide the combination of a limited number of meaningless sounds (phonemes) in a variety of ways to generate new meaning [1,2]. In a simple example, the phonemes /k/, /æ/ and /t/ can be rearranged in different ways to create the words *cat* [kæt], *act* [ækt] or *tack* [tæk] [1]. Alternatively, the phoneme /k/ from the word *cat* can be eliminated to create the word *at* [æt], with the first position (i.e., presence or absence of the phoneme /k/) representing a phonemic contrast that generates the differentiation in meaning [3]. In all four arrangements, the meaningless phonemes maintain their acoustic identity across words, and this, paired with the arbitrary relationship between phoneme structure and word meaning, results in words with shared phonemes having distinct semantic content [4]. Such phoneme structure is a basic ingredient of word generation in human language, and when combined with the rules governing assemblages of meaningful words (a syntactic layer), provides much of language’s generative power [5–7]. Despite the crucial role that phoneme structure plays in language, little is known about how such a capacity might have evolved [8–11]. Whilst comparative data from animal communication systems can elucidate early forms of language components, data demonstrating the critical rudiments of phoneme structures outside of humans is lacking.

Evidence that animals can employ a basic syntactical layer of language in their communication system has been provided in nonhuman primates. For example, Campbell’s monkeys (*Cercopithicus cambelli*) produce two predator-specific alarm calls that are each modified in a predictable way into more general disturbance calls upon addition of the same suffix [12,13]. However, because the constituent calls are themselves meaningful (with the suffix carrying an abstract meaning in this case [14]), this, and equivalent findings [15,16], do not exemplify phoneme structure. Several candidates of phoneme-like structures in nonhuman animals have been proposed, but defining features are either lacking or have yet to be demonstrated [8,11,17]. One set of contenders comes from the songs of birds and mammals, in which meaningless elements are combined to create complex, higher-order structures [11,18,19]. However, experiments investigating behavioural responses to element reorganisation within songs are either lacking [18–21] or have not shown that such reorganisation confers a qualitative change in contextual meaning [22–24]. Another set includes calls produced in movement and alarm contexts. For example, parid birds can produce variable vocal sequences of apparently meaningless acoustic elements. However, in these cases, although call elements are commonly repeated or omitted, the required association between sequence structure and qualitative changes in informational content has not been demonstrated [25–29].

**Fig 1:**
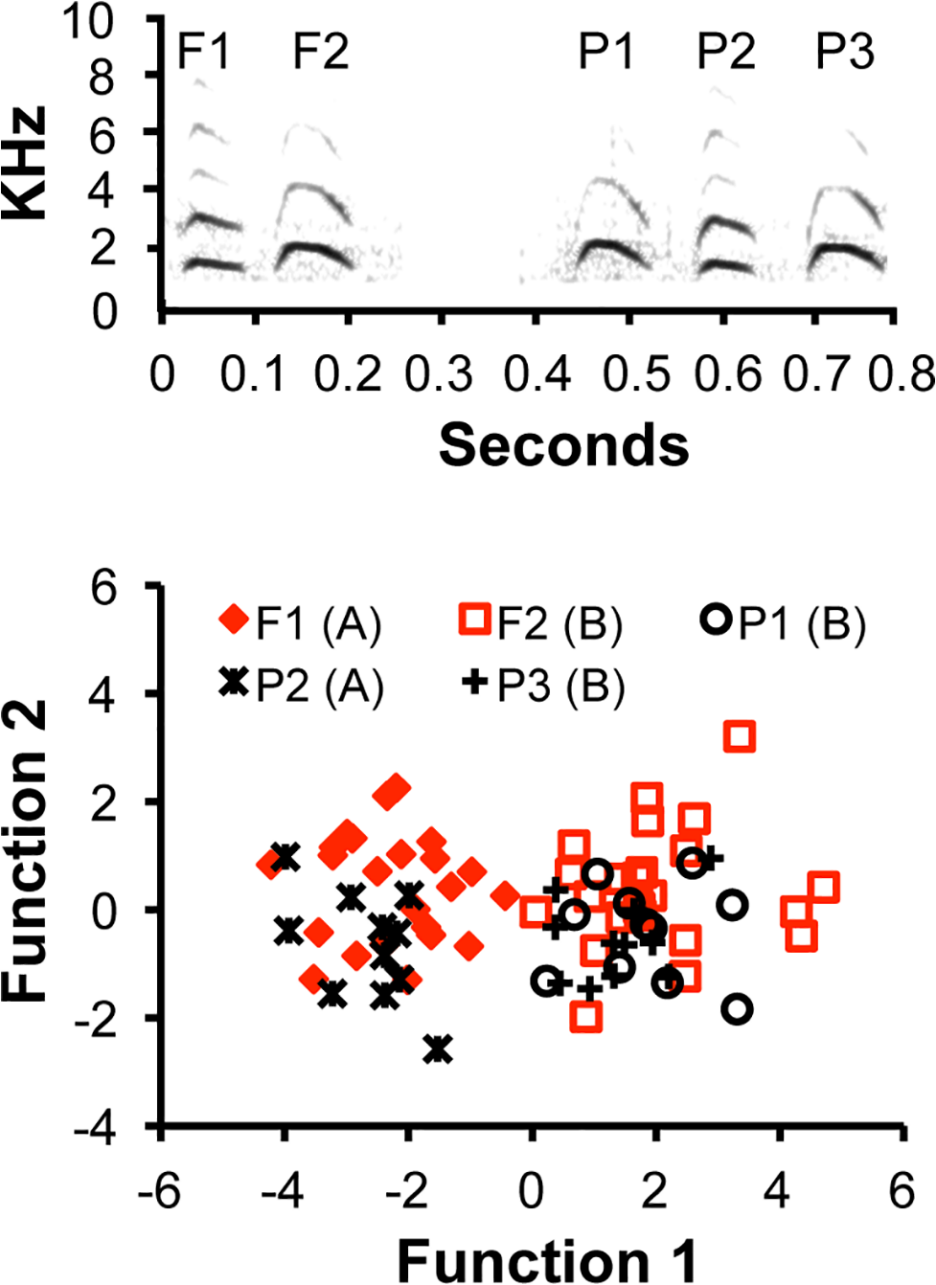
Flight and prompt call structure. (A) Spectrogram of double-element flight call (sequence F1 F2) and triple-element prompt call (sequence P1 P2 P3), taken from different individuals and groups. (B) Discriminant Function Analysis (DFA) output: function 1 explains 95% of the variance in element structure and primarily describes frequency range; function 2 explains the remaining 5% of variance and describes the contrast between start/end frequency (positive loadings) and frequency range (negative loadings) (Materials and Methods; S1 Text; S1 Table; S2 Table). F1 could not be reliably discriminated from P2 (34% errors: *T*_32_ = 1.4, *p* = 0.2) and nor could F2, P1, and P3 be discriminated from each other (27%–32% errors: F2 versus P1: *T*_32_ = 0.7, *p* = 0.4; F2 versus P3: *T*_32_ = 1.4, *p* = 0.2; P1 versus P3: *T*_20_ = 0.2, *p* = 0.8), but F1/P2 could be easily distinguished from F2/P1/P3 (all 0% errors) (*T*_32–44_ = 14.1–22.9; all *p* values < 0.001; S3 Table). Accordingly, flight calls and prompt calls follow *AB* and *BAB* construction, respectively.

Using acoustic analysis, natural observations, and controlled playback experiments we provide evidence for rudimentary phoneme structure in the calls of chestnut-crowned babblers (*Pomatostomus ruficeps*) (see Materials and Methods), a 50 g, highly social, cooperatively breeding bird [30,31]. Observations over the past 10 years suggest that the repertoire of adult chestnut-crowned babblers consists of at least 15 discrete, context-specific vocalizations, of which three pairs appear to share sound elements, with the reused elements in each case being restricted to a specific pair of calls [32]. Here, we specifically focused on a single pair: a double-element call produced during flight (flight calls, elements F1 and F2) and a triple-element call produced during nestling provisioning [33] (prompt calls, elements P1, P2, and P3) (Fig 1A). Importantly, the constituent elements within these calls appear to be contextually meaningless. For example, none of the elements is used as an individual call in isolation, suggesting that none can function to confer contextual information. Additionally, because none is used in combination with other call types, they cannot clearly operate to modify calls in a predictable way, as would be required of affixes [13]. First, we establish, using acoustic analyses, that the two calls comprise statistically equivalent acoustic elements. Second, we present natural observations showing that the two calls are context-specific, a prerequisite of reliable information transfer in animals. Finally, playbacks of natural, switched-element, and artificial calls in a standardised aviary environment confirm that the call elements are perceptibly equivalent and that element addition/elimination at one position creates a phoneme-like contrast, yielding the functional changes in meaning.

## Results

### Acoustic Analysis

Acoustic analyses were conducted to test whether prompt and flight calls are composed of statistically indistinguishable acoustic elements. To avoid problems of pseudo-replication arising from using calls of genetic relatives within groups [34], we analysed a single flight call and a single prompt call per group recorded (*n* = 23 flight, 11 prompt calls). Five parameters were extracted from the fundamental frequency of the resulting 79 elements: start and end frequency, frequency range, time to peak frequency, and element duration (S1 Text, S1 Table, and S2 Table). A Discriminant Function Analysis (DFA) demonstrated that the five elements across the two calls comprised just two independent acoustic structures (Fig 1B). Mahalanobis distances generated from the DFA revealed that F1 and P2 could not be reliably distinguished and neither could F2, P1, and P3 (all *p* values > 0.2), but that F1 and P2 could be distinguished easily from F2, P1, and P3 (all *p* values < 0.001) (Fig 1B and S3 Table). Thus, the two calls appear to comprise the same two distinct elements, with flight and prompt calls displaying *AB* and *BAB* patterns, respectively.

### Natural Observations

Natural observations were conducted to quantify the context in which flight and prompt calls are produced. Natural flights were accompanied by flight calls in 274 of 450 observations (61%; *n* = 6 groups, 1 h/group), with all flights being short, low, and easily quantified. Similarly, hand-held releases following capture induced flight calls in 58 of 90 occasions (64%, *n* = 24 groups). No prompt calls were recorded in either set of observations, and flights/releases lacking flight calls were either silent or associated with alarm calls in response to observer presence. Finally, recordings from within nests in conjunction with automated nest entry-exit recorders revealed that 62% flights to/from nests were accompanied by flight calls (*n* = 140 visits, 7 groups) but rarely prompt calls (0.08% of nest visits), while 70% of nestling provisioning events were associated with prompt calls (*n* = 140 visits, 7 groups) and rarely flight calls (0.03% of nest visits). Additionally, in 97% of nest visits in which both flight and prompt calls were recorded, individuals used only flight calls travelling to/from the nest and only prompt calls within nests (*n* = 60 visits, 7 groups). Thus, flight and prompt calls are highly context-specific, with the former maintaining group cohesion during movement [35] and the latter increasing the efficiency of food transfer to offspring by stimulating begging [33].

### Playback Experiments

**Fig 2:**
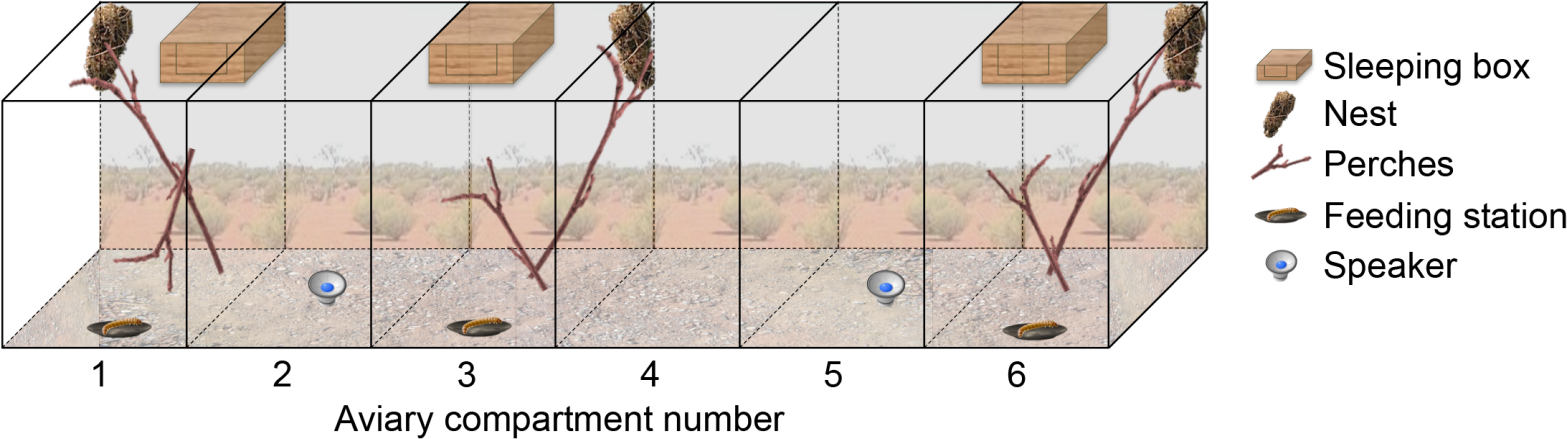
Schematic of aviary setup. The aviary consisted of six compartments: the back comprised metal meshing (1 cm^2^), allowing the birds an outside view; the two sides were made of aluminium; and the front was specially designed perspex, allowing a one-way view from outside to inside. Occupied compartments contained natural perches, foraging substrate, a feeding station, babbler nest, and sleeping box, while unoccupied compartments contained the playback apparatus. Babbler nests are large (~45 x 30 cm), dome-shaped, with 6 cm diameter entrance hole, and robust. Babblers spent most of their time at mid-height; in all cases, relative to the speaker, birds had to look behind and up to look at the nest. Single birds used compartment 3 (*n* = 2), pairs of birds used compartments 1 and 3 (*n* = 1 pair) and trios used compartments 1, 3, and 6 (*n* = 4 trios) (S2 Text).

To verify experimentally that flight and prompt calls are context specific and are generated from rearrangement of the same acoustic elements, we performed playback experiments on 16 birds captured from 7 groups during periods of breeding. Each of the 16 birds received six playback trial-sets presented in a randomised order. Behavioural responses to two natural, two switched-element and two artificial calls were recorded in aviary compartments (2 x 2.5 x 2 m l x b x h) containing natural perches, foraging substrate, a view to the outside, and a recently used babbler nest (30x45 cm dome-shape, 6 cm diameter entrance) (Fig 2). The playback speaker was positioned out of view in a neighbouring compartment; birds had to look perpendicular to the speaker to look outside the aviary and in the opposite direction to look at the nest (S2 Text). Given our natural observations, we predicted flight calls would elicit increased observations to the outside and increased movement in anticipation of an incoming bird, while prompt calls would provoke greater nest attentiveness. Combined, these three behaviours comprised 61% of the activity budget in each trial (SD [standard deviation] = 23%; correlation coefficients among these behaviours ranged from +0.1 to -0.3, indicating that time spent in one activity did not preclude time available for another).

**Fig 3:**
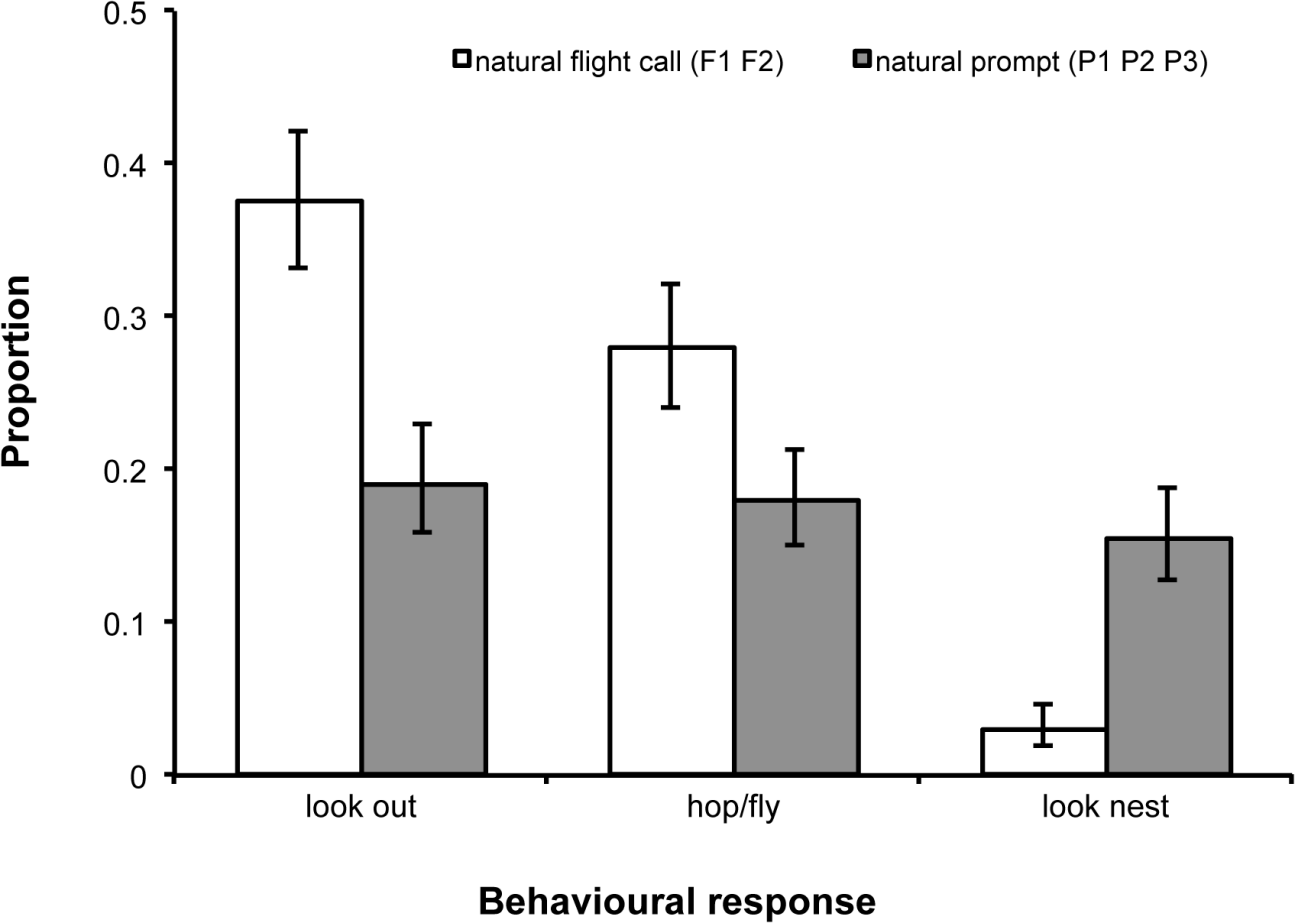
Responses to natural playbacks. Proportion of time spent engaged in three behaviours of functional relevance differed significantly during the playbacks of the two call types (see text). Figure shows back-transformed predicted means (± standard error [SE]) generated from three Generalized Linear Mixed Models (GLMM), in which the time engaged in each of the three activities (looking out of the aviary, in movement [hopping/flying], and looking at the nest) were fitted as three independent response terms. In each case, response terms were fitted to a binomial error structure with logit link function, time spent in camera view was fitted as the binomial denominator, call type (natural flight versus natural prompt) was fitted as a two-level factor, and individual identity nested within group identity were fitted as random terms.

Compared with natural prompt calls, natural flight call playbacks were associated with a 49% increase in the proportion of time spent looking outside (Generalized Linear Mixed Model [GLMM]: χ^2^_1_ = 11.8, *p* < 0.001) and a 36% increase in time spent hopping/flying between perches (χ^2^_1_ = 6.5, *p* = 0.02). By contrast, during natural flight call playbacks, individuals spent 81% less time looking at the nest (2% of monitoring time) than during prompt call playbacks (15% of time) (χ^2^_1_ = 11.6, *p* < 0.001) (Fig 3). Together, these results confirm the two calls are distinct and encode perceptible, context-specific information.

**Fig 4:**
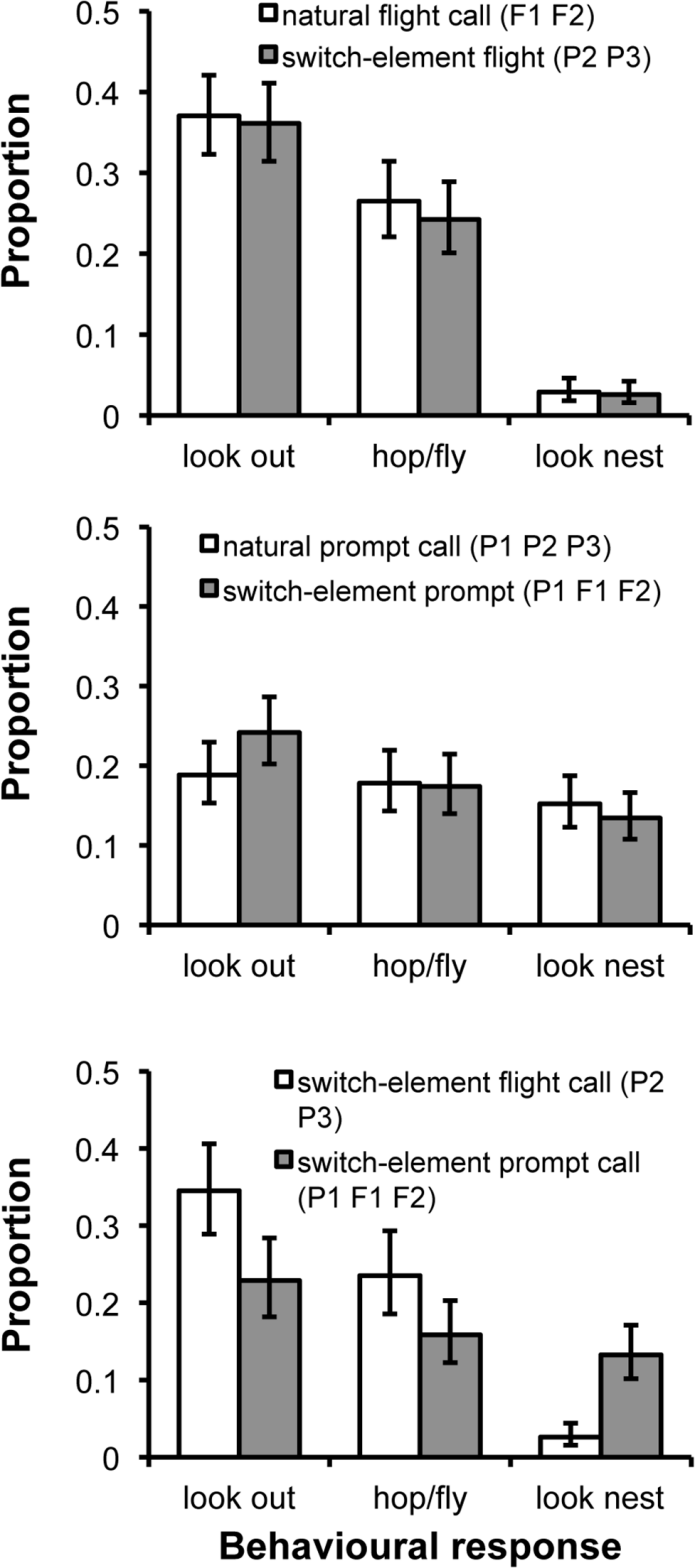
Responses to calls with and without reciprocal element exchange. Behavioural responses of functional relevance remained similar between (A) natural flight calls and switched-element flight calls comprising prompt call elements (natural versus switched-element comparisons: look out: χ^2^_1_ = 0.02, *p* = 0.9; hop/fly: χ^2^_1_ = 0.2, *p* = 0.6; look nest: χ^2^_1_ = 0.03, *p* = 0.9) and (B) natural prompt calls and switched-element prompt calls using the two flight call elements (natural versus switched-element comparisons: look out: χ^2^_1_ = 0.2, *p* = 0.6; hop/fly: χ^2^_1_ = 1.3, *p* = 0.3; look nest: χ^2^_1_ = 0.01, *p* = 0.9). (C) Behavioural responses to switched-element flight and switched-element prompt calls differed significantly or showed a non-significant tendency to do so (switched-element flight versus switched-element prompt call comparisons: look out: χ^2^_1_ = 5.7, *p* = 0.02; hop/fly: χ^2^_1_ = 3.2, *p* = 0.09; look nest: χ^2^_1_ = 10.0, *p* = 0.002). Analyses were conducted as above (Fig 3) except that in (A) and (B) (which are shown separately for clarity) stimulus type (natural versus switched-element) and its interaction with call type were added as additional fixed effects, while in (C), natural flight and prompt calls were replaced with switched-element ones. All figures show back-transformed predicted means (±SE).

To test whether unmeasured acoustic variation dissociates the two calls [15], we played back switched-element versions of both calls to all 16 birds by generating flight calls from prompt elements P2 P3 and prompt calls from elements P1 F1 F2. The proportion of time birds spent engaged in the three behaviours of functional relevance were statistically equivalent between natural and switched-element flight calls (GLMM: all *p* values > 0.6; Fig 4A) as well as between natural and switched-element prompt calls (all *p* values > 0.3; Fig 4B). Additionally, there were no significant interactions between call type (flight versus prompt) and whether or not calls were natural or switched-element on behavioural responses (GLMM: all *p* values > 0.4). The absence of such interactions generated differences in behavioural responses to switched-element flight versus switched-element prompt calls of similar magnitude to those found in comparisons of natural calls (see Fig 4C versus Fig 3). Compared with switched-element prompt calls, switched-element flight calls were associated with 33% more time looking out, 33% more time in-movement, and 80% less time looking at the nest. Accordingly, it is improbable that any unmeasured acoustic differences between the elements of flight and prompt calls are responsible for the distinct responses, reinforcing our acoustic analyses that the calls comprise the same sound elements.

The results above suggest that the meaning-differentiating element between the two calls is P1. Before a phonemic-like system can be supported, two other interpretations require testing. First, element P1 might, by itself, be responsible for generating the contextual information carried by the prompt call, in which case, our results could be more akin to a syntactic, rather than phonemic, communicative system [12,13]. Second, the differences in response to flight calls versus prompt calls might arise from their differences in element number [36]. In this case, our results could represent stimulus intensity effects (triple-element prompt versus double-element flight call) or priming effects [12] (any acoustic element preceding a flight call results in a prompt-type response). To test these alternative interpretations, we presented two artificial stimuli to the 16 birds: element P1 alone and *CAB*, with the latter representing call elements P2 P3 (i.e., *AB*) preceded by an element (*C*) from chatter calls, a common call naturally repeated in mixed-element bouts and associated with excitement [32].

**Fig 5:**
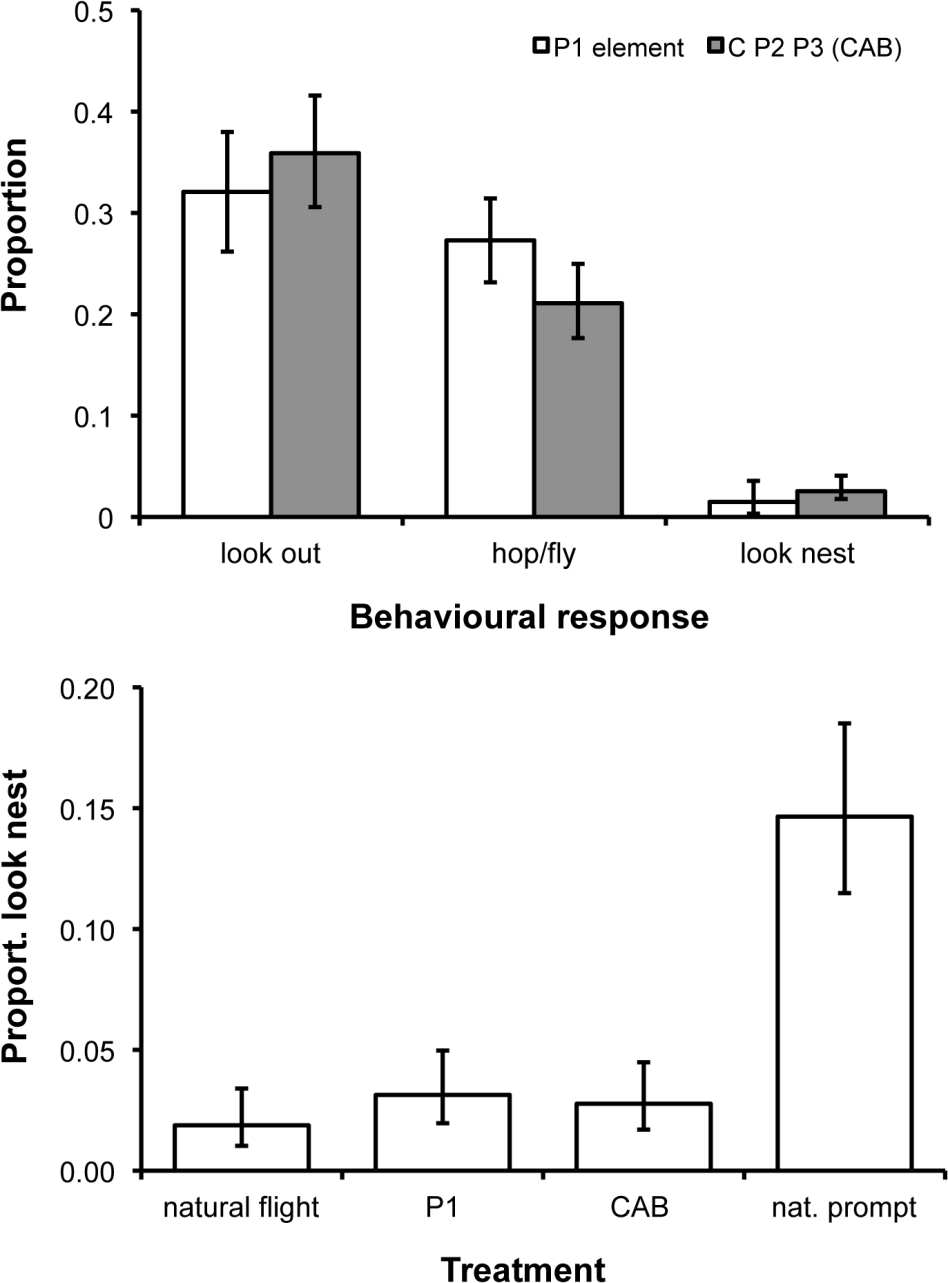
Behavioural responses to artificial calls. (A) During P1 element and *CAB* playbacks, individuals spent comparable proportions of time looking out (χ^2^_1_ = 0.2, *p* = 0.6), in movement (χ^2^_1_ = 1.8; *p* = 0.2), and looking at the nest (χ^2^_1_ = 0.5; *p* = 0.5). (B) Neither P1 nor *CAB* playbacks provoked an increase in nest-attentiveness over natural flight calls, leading to proportions of time spent looking at the nest during these trials being substantially lower than those generated during natural prompt calls (χ^2^_3_ = 25.4; *p* < 0.001). Figures show back-transformed predicted means (±SE) generated from GLMM analyses as outlined in Fig 3 (A) or in which the two-level factor was replaced with a four-level factor (B).

These two artificial stimuli elicited similar behavioural responses (all *p* values > 0.2; Fig 5A). First, they both generated relatively high look out and movement responses. One explanation lies with the fact that each is unnatural: impossible vocal scenarios have been shown to increase attentiveness behaviour in other contexts [37,38]. In support, separate analysis of the proportion of time spent looking around the aviary showed that general attentiveness behaviour during natural flight playbacks (mean ± SE = 16% ± 4%) was 36%, 47%, and 48% lower than during playbacks of *CAB*, P1, and natural prompts, respectively (GLMM: χ^2^_3_ = 10.6, *p* = 0.01). Second, and more crucially, neither the P1- nor the CAB-stimulus elicited a hint of an elevated response in nest-attentiveness (Fig 5B). Like the flight call, P1 element and *CAB* playbacks were both associated with ca. 80% reductions in nest-attentiveness behaviour over natural prompt calls (Fig 5B). That neither the P1 element alone nor *CAB* elicits any increase in nest-attentiveness confirms that (a) P1 does not carry any nest-associated information in isolation and (b) differential nest-attentiveness responses to flight and prompt calls are not derived from either stimulus intensity or priming effects. Thus, it is the presence or absence of element P1 from the P2 P3 (or F1 F2) call sequence that appears integral to generating the qualitatively distinct meaning carried by the two calls.

## Discussion

Phoneme structure represents a critical component of the vast lexicons in human languages, but a lack of suitably comparable evidence in animals has hindered our understanding of candidate selection pressures on, and early forms of, phoneme structure. Two related hypotheses have been proposed to explain the emergence of phonemic systems; both advocate a role of selection acting on increasing the capacity of vocal communication beyond that currently possible under an existing vocal repertoire. The here-named “enhanced-perception hypothesis” proposes that stringing together existing sounds in new ways reduces perception errors over the generation of new, but similar, sounds [39–41]. By contrast, the “vocal-constraints hypothesis” proposes that when the generation of new sounds is constrained [42], reusing pre-existing sounds in new combinatorial forms can provide an alternative solution to increasing communicative output [15,16]. Testing the predictions arising from these hypotheses represents a major challenge because human languages are generally too derived to address the pressures selecting for their emergence. Additionally, testing whether animals make perceptual mistakes for sounds that do not exist or are vocally constrained will be rarely feasible. A necessary first step in elucidating the pressures selecting for, and early forms of, phonemic structure is to address whether animals possess the capacity for generating functionally distinct vocalizations by rearranging contextually meaningless elements, and how such rearrangements are manifest.

Here, using acoustic analyses, natural observations, and playback experiments, we reveal that chestnut-crowned babblers use two acoustic elements (*A* and *B*) in different arrangements to create two functionally distinct vocalizations: flight calls (F1 F2, or *AB*) and prompt calls (P1 P2 P3, or *BAB*). The meaning differentiation between the two calls is not a result of the different number of elements or priming effects, but specifically the presence or absence of P1 (element *B*) at the head of the same call sequence. The fact that element P1 is both contextually meaningless on its own and meaning differentiating when used in combination with elements P2(F1) and P3(F2) signifies a phoneme-like contrast, with element *B* used in this position likely representing a phoneme-equivalent. To our knowledge, this is the first demonstration that animals have the basic capacity to use phoneme-like contrasts to derive qualitatively new meaning, a basic component of phoneme structuring. However, whether or not our results can also be interpreted as providing evidence for more advanced forms of phoneme structuring in an animal depends on two critical features.

First, in human languages, phoneme structure has potentially boundless generative power: the sum of derivable information is substantially greater than the number of its phonemic parts [1]. In contrast, the babbler vocal system that we describe is strictly bounded in its generative nature (i.e., two elements generate only two distinct calls). Part of the difference in human versus any nonhuman phonemic system will inevitably arise from vast differences in cognitive capacity [9]. Notwithstanding, cognitive capacity alone does not appear to be sufficient to explain differences in phonemic complexity and boundedness. For example, the sign language of the Al Sayyid Bedouin, an emerging language shared by deaf and hearing people of a small Israeli village, has been shown to have a fully functional and productive syntactic layer, but is so far characterized by only one phonological form [43,44]. Thus, when a phonemic layer emerges, even in human language, it appears initially to be finite and strongly bounded. This evidence suggests that the use of phonemic structure in communication should not be defined a priori by its complexity or boundedness, for it is likely that all phonemic systems evolve from simple beginnings like the one we describe here.

Second, the level of phonemic complexity used by babblers depends on the number of phoneme-equivalent entities in use. For example, whilst babblers generate a phonemic contrast by inserting the phoneme-like entity P1 before P2(F1) and P3(F2), whether or not P2 and F1 or P3 and F2 also represent phoneme-equivalent entities in the linguistic sense is equivocal. Unlike combinatoriality based on affixation rules or the generation of idioms, in which constituent parts have meaning [12,16], definitively testing whether all sound elements within call sequences of animals are contextually meaningless, and yet individually perceptible and meaning-differentiating, will be a major challenge. This is because any sound uttered by a conspecific can lead to a behavioural response irrespective of any perception of contextual meaning [38], and their limited vocal repertoires preclude investigation of whether distinct functional meaning is derived from the same meaningless elements in multiple different arrangements. A key component in discerning whether F1/P2 and F2/P3 are also phonemic depends on whether they represent a compound of two discrete elements, perceptible independently (i.e., *A* and *B*), or a holistic unit (i.e., *AB*). That the *B* element is phonemic in position P1 hints that *AB* is reducible, and hence F1/P2 and F2/P3 are probably also phoneme-like. However, this is an untested hypothesis at this stage, and we do not wish to speculate on whether chestnut-crowned babblers use more advanced forms of phoneme structure, beyond the identified use of a simple contrast, as part of their communication system.

Either way, we propose that the bounded use of phoneme-like contrasts in the vocal system of chestnut-crowned babblers represents a simple precursor of phoneme structuring that can elucidate how early forms of phonemic systems might emerge. For example, our results lead to the hypothesis that the addition or elimination of elements, i.e., basic phonemic contrasts (e.g., /kæt/ versus /æt/), might represent a simpler evolutionary step than complete element rearrangement (e.g. /kæt/ versus /tæk/), due to its reduced structural complexity. However, generating distinct contextual meaning through the former rather than latter process is likley to be more prone to perception errors, because it results in higher acoustic similarity. That babblers have opted for the more error-prone means of generating functionally distinct vocalizations, and done so by adding or eliminating a common element, is more supportive of a vocal-constraints hypothesis [15,16] than an enhanced-perception hypothesis [39–41]. Limiting the use of phonemic contrasts to short-range calls used in low-urgency, social contexts might be one way of reducing perception errors and mitigating associated costs when vocal constraints are operating.

In conclusion, the salient message here is that the basic capacity to generate qualitatively new meaning from rearranging contextually meaningless elements appears to exist outside of humans. One explanation is that for vocally constrained, highly social species, such as chestnut-crowned babblers, evolving new meaning by rearranging existing sounds offers a faster route to increasing communicative output than evolving new sounds. We hypothesise that reusing acoustic elements has facilitated the emergence of phoneme-like contrasts, which potentially drove sensitivity to phoneme structure or “phonemic awareness” in receivers [45,46]. The capacity to recognise vocalizations as sound constructs composed of smaller, meaningless elements, instead of a holistic unit, may have been the first step in the emergence of the elaborate phonemic systems seen in human languages. Further experiments are now required to determine exactly how babblers compute and perceive the elements from the two calls. More generally, further evidence for the use and manifestation of phonemic systems in animals is required; we propose that such systems will be most operant in the short-range communication of vocally constrained, social animals.

## Materials and Methods

### Study Site and Species

Ethics approval was provided by Macquarie University, Sydney, Australia (Number ARA 2013/025). The study was conducted on a population of wild, unhabituated chestnut-crowned babblers at the Fowlers Gap Arid Zone Research Station in far western New South Wales, Australia (141°42´E, 31°06´S). The population has been studied intensively since 2004. The habitat is characterised by low, open, chenopod shrubland, with trees largely confined to short, linear stands in drainage zones. Chestnut-crowned babblers (~50 g) are ground-foraging, weak-flying, and highly cooperative. During non-breeding they live in groups of 3–23 (mean ≈ 10) individuals, which then partially fragment into 1–4 units of 2–15 individuals (mean ≈ 6) for breeding. Non-breeders associate with those breeders to which they are most related and have substantial effects on their breeding success, primarily by reducing nestling starvation and facilitating additional reproductive attempts by the breeders. Further details on habitat and babbler socio-ecology are provided elsewhere [30,31,33,47–49]. All statistical analyses were performed in Genstat Release 17 (VSN International Ltd, Hemel Hempstead, UK, 2014). Data used in analyses and figure generation can be found in Dryad: http://dx.doi.org/10.5061/dryad.082v2 [50].

### Context of Flight and Prompt Calls

We quantify the use of flight and prompt calls in three different contexts. First, in 2010, we used focal observations (1 h each on six groups from a distance of ~25 m) to determine the frequency with which the two calls are uttered during natural flights (*n* = 450 flights). Second, in 2011, we used a Fostex FR2-LE and wind-shielded Sennheiser ME67 shotgun microphone to record the vocalizations uttered during manual releases from cloth bags following capture (observers under a bedsheet; *n* = 90 releases from 25 groups). Third, in 2012, we fitted Yoga EM-400 mini tie-clip microphones to the wall of nests during nestling provisioning and recorded vocalizations using an Olympus LS-10 PCM or Fostex FR2-LE. To quantify the use of flight and prompt calls during flights to and from the nest, as well as during provisioning within the nest, we coupled the above nest-recording system with a transponder system, allowing the timing of bird entrances and exits to and from the nest to be determined [30,33,47]. Briefly, by inserting transponder tags (2 x 12 mm) into the flanks of the birds and fitting an antenna around the nest entrance linked to a TROVAN decoder, we were able to determine the use of the two calls within 5 s of entering and exiting the nest. Nest recordings were made 7 A.M.–4 P.M., in August–October, when broods were 1–12 days old. The first 20 nest visits within recording periods were used to quantify call-use at seven nests (time taken for 20 visits: 68–401 min.; *n* = 140 visits).

### Acoustic Extractions and Statistical Analysis of Natural Calls

To quantify the resemblance among the five elements within and between double-element flight calls and triple-element prompt calls, we selected a single flight and prompt call recorded from each group during releases and nest recordings (sampling frequency of 44.1 kHz, 16 bits). Calls were selected randomly from those exhibiting no obscuring vocalizations, high signal-to-noise ratio and low background noise, and blindly with respect to the analyses. The elements of such calls (*n* = 23 double-element flight calls and 11 triple-element prompt calls) were then extracted using Raven Pro, version 1.4 (Bioacoustics Research Program, Cornell Lab of Ornithology, Ithaca, NY, 2011). Five parameters were extracted from the fundamental frequencies of the five elements in the two call types (start and end frequency, time to peak frequency, frequency range, and element duration). All parameters were normalised when necessary and then centred to the mean and standardised by dividing the centralised mean values by 2-fold their standard deviation, allowing direct comparison of the importance of each parameters within and between models [51]. Two of the ten possible correlation coefficients among the five parameters were significantly positive: element length and frequency range (*r*_*p*_ = 0.65, *p* < 0.001); and start and end frequency (*r*_*p*_ = 0.38, *p* = 0.002) (S1 Table). Preliminary Analyses of Variance (ANOVA) (S1 Text) showed that time to peak frequency was statistically invariant across the five elements, and this was also the case for element durations after controlling for its correlation with frequency range. By contrast, start and end frequency, as well as frequency range, all varied between the elements, and for start and end frequency, this was the case after controlling for their correlation with each other. The three element parameters found to have significant independent effects on element structure (frequency range, start frequency, end frequency) were then used in a Discriminant Function Analysis (DFA) to determine acoustic similarity (S2 Table; S3 Table).

### Playback Experiments: Test-Subject Selection and Housing

All playback experiments were conducted on wild birds captured in mist-nets on their territories during periods of breeding. Test subjects were chosen randomly from all adult birds captured (>6 months old), excluding the breeding female, without regard for sex and age. Depending on a test subject’s group size, 1–3 individuals were removed (<30% of group members); resulting in 16 individuals from 7 different groups being tested. Removed birds were transported the 1–5 km immediately by car to aviaries on site at Fowlers Gap and released into separate aviary compartments (Fig 2). The aviaries consisted of six single compartments each of 2 m long, 2.5 m deep and 2 m high. Birds were housed singly, and each fed 20 mealworms every 2–3 h of daylight, delivered through a tube into each aviary compartment, of which 8–15 were typically consumed per bout. Birds gained a mean of 0.65 g (range = -3.1 to +4.8 g) during their time in the aviary; all birds were released near their original group less than 48 h after initial capture, and were accepted back into their group without any signs of aggression [49].

### Playback Experiments: Rationale, Call Recordings, and Playback Protocol

Our primary objective in this study was to test whether babblers used a phonemic contrast to generate qualitatively new information. For purposes of experimental rigour and analytical clarity, we chose a fully balanced design, with each bird being presented with the full set of selected playback stimuli. The drawback of presenting multiple stimuli to the same birds lies in the risk of habituation, leading to the generation of ambiguous results. For this reason, we decided to limit the number of playback trials to the absolute minimum number required to test for a phonemic contrast (i.e., six).

Our rationale for the six playback stimuli chosen was as follows. First, given the primary focus, the critical experiments needed to include natural and switched-element versions of both calls (i.e., amounting to four playback conditions). Second, because the acoustic analyses suggested that the only difference between the two calls derives from P1 in prompt calls, we deemed it key to test whether this element alone partially contributes to the overall meaning of the prompt call by eliciting an increased nest-attentiveness response compared with the flight call. If this were the case, we would have evidence of something more akin to a syntactic than phonemic system. Finally, because flight and prompt calls comprise two and three elements, respectively, we thought it essential to test for an influence of this difference in generating variation in nest attentiveness. We chose a stimulus including C1 P2 P3 because, again, we deemed it most informative for the key aim to manipulate the one element that differs between the two calls (i.e., P1). The C1 element was taken from chatter calls: a common multi-element call uttered in bouts of several seconds in contexts of excitement or alarm [32]. The single C1 element was of comparable duration to the replaced P1 element.

The calls used in the playback experiments were obtained from natural recordings at the nest of six groups. In each case, a Sennheiser directional microphone (ME66/K6) connected to a Marantz solid-state recorder (PMD660, sampling frequency 48 KHz, 24 bits) was positioned within 1 m of a nest. Playbacks, including the construction of artificial calls (see below), were created with Adobe Audition CC (Version 6 Build 732, Adobe Systems), selecting high-quality calls (as above). Of the high-quality calls obtained, a single double-element flight call, triple-element prompt call, and a single element of the mixed-element chatter call were selected from each of the six groups (*n* = 18 calls). For each of the six groups from which recordings were obtained, the set of six playback stimuli were created, with each set including a natural flight call (F1 F2), a natural prompt call (P1 P2 P3), a switched-element flight call (P2 P3), a switched-element prompt call (P1 F1 F2), a P1 element stimulus (P1), and a triple-element stimulus (C1 P2 P3). In all cases, except for one, birds were tested with a new call-set played in randomized order, and birds never received a call-set from their own group. When elements for the generation of artificial and control calls were added and/or replaced, it was ensured that inter-element distance and amplitude matched the original call (Fig 1A). During each playback, a stimulus was repeated six times randomly distributed over 3.2–3.6 s; a break of at least 10 min was given for focal individuals to resume pre-stimuli behaviour before the initiation of another stimulus.

Playback experiments were conducted on the day following capture. Individuals of the same group were tested simultaneously with the same playback-set, but they were always housed separately and could not see each other (Fig 2 and S2 Text). Nevertheless, birds tested simultaneously could influence each other’s behaviour if they reinforced (or countered) the playback experiment with their own vocalizations. This was not the case. In the 420 seconds of the playback experiment, not a single prompt call was uttered, and only 24 flight calls were given by the 14 individuals tested simultaneously, leading to a flight call rate of 0.28 per bird per 10 s trial. Additionally, of these 24, only ten were produced during natural or artificial flight call playbacks, all by two of the five groups. Finally, adding whether or not a flight call vocalization was uttered during the playbacks never impacted the explanatory power of the models (all *p* values > 0.8).

During testing, individuals were recorded using digital Sony handycams (HDR-CX220 and HDR-CX160) through a viewing hole to increase image clarity. Visual recordings of 10 s from playback onset were analysed frame by frame using Adobe Audition CC (Version 6 Build 732, Adobe Systems), with time (s) spent in camera view (mean = 9.4 s, range = 10–6 s), looking at the nest, looking outside (i.e., towards mesh wall), and in movement (hopping or flying) representing the primary parameters of interest, although general looking around behaviour was also recorded. Marker lists created in Adobe Audition were extracted into txt-files by using CueListTool (Version 1.7), and rates were calculated.

### Playback Experiment: Statistical Analyses

Analyses of behavioural data arising from the playback experiments were conducted using Generalized Linear Mixed Models (GLMM), in which the time spent engaged in a given behaviour was fitted as the response term and the total amount of time spent in camera view was fitted as the binomial denominator. Explanatory terms included natural flight and natural prompt calls only (Fig 3); call type (flight or prompt), trial type (natural or switched-element), and their interaction (Fig 4A and 4B); switched-element flight and switched-element prompts calls only (Fig 4C); and element P1 and *CAB* stimuli only (Fig 5A) or as a four-level factor with natural flight and natural prompt calls (Fig 5B). Additionally, the time spent in view was fitted as a covariate in a single movement analysis (Fig 3). In all GLMM analyses, individual identity nested within group identity were fitted as random terms. Doing so served two purposes: (1) it blocked the analyses by individual, effectively generating a more powerful repeated measures statistical design, and (2) it accounted for any lack of independence arising from testing birds from the same group simultaneously with the same playback stimuli. Regarding this potentially important latter issue: in all analyses, group identity was non-significant (all *p* values = 0.4–0.9), indicating that there was statistically equivalent variation in individual responses from the same group to the same playback stimuli as there was in individual responses from different groups to different playback stimuli.

## Supporting Information

S1 Table Correlations among call-element parameters. (DOCX)

S2 Table Discriminant Function Analysis (DFA) statistical output of call-element parameters. (DOCX)

S3 Table Mahalanobis D-squared distances generated from DFA. (DOCX)

S1 Text Statistical analysis of call-element parameters. (DOCX)

S2 Text Aviary set-up during playbacks. (DOCX)

## Abbreviations:

DFA: Discriminant Function Analysis
GLMM: Generalized Linear Mixed Model
