# Supplementary material for: Experimental Evidence for Phonemic Contrasts in a Nonhuman Vocal System
Source: PLoS Biol. 2015 Jun 29;13(6):e1002171. doi: 10.1371/journal.pbio.1002171 (PMC4488142; doi:10.1371/journal.pbio.1002171)
Supplement: S1 Table — (DOCX) [file pbio.1002171.s001.docx]

Experimental Evidence for Phonemic Contrasts in a Nonhuman Vocal System: Engesser et al

**Supplementary Table**

S1 Table. Pearson correlation coefficients (*r_p_*) and significant values in parentheses for the five acoustic parameters measured from the two calls.

|  | EL | SF | EF | FR | TPF |
| --- | --- | --- | --- | --- | --- |
| Element length (EL) | - |  |  |  |  |
| Start frequency (SF) | -0.10 (0.53) | - | - | - |  |
| End frequency (EF) | -0.087 (0.55) | 0.38 (**0.002**) | - | - |  |
| Frequency range (FR) | 0.65 (**<0.001**) | 0.12 (0.37) | 0.067 (0.95) | - |  |
| Time peak frequency (TPF) | 0.30 (0.69) | -0.24 (0.11) | 0.30 (0.064) | -0.23 (0.15) | - |
