# Supplementary material for: Experimental Evidence for Phonemic Contrasts in a Nonhuman Vocal System
Source: PLoS Biol. 2015 Jun 29;13(6):e1002171. doi: 10.1371/journal.pbio.1002171 (PMC4488142; doi:10.1371/journal.pbio.1002171)
Supplement: S2 Table — (DOCX) [file pbio.1002171.s002.docx]

Experimental Evidence for Phonemic Contrasts in a Nonhuman Vocal System: Engesser et al

**Supplementary Table**

S2 Table. Discriminant function output, based on the inclusion of frequency range, start frequency and end frequency.

|  | DF1 | DF2 |
| --- | --- | --- |
| Eigenvalue | 4.66 | 0.24 |
| % variance | 95 | 5 |
| Statistics (*χ*^2^) | 145.6, *DF*=12, *P*<0.001 | 17.3, *DF*=6, P=0.008 |
| Standardised vector loadings of traits | |  |
| Start frequency | 0.15 | 0.68 |
| End frequency | 0.16 | 0.82 |
| Frequency range | 0.78 | -0.62 |
