# Supplementary material for: Experimental Evidence for Phonemic Contrasts in a Nonhuman Vocal System
Source: PLoS Biol. 2015 Jun 29;13(6):e1002171. doi: 10.1371/journal.pbio.1002171 (PMC4488142; doi:10.1371/journal.pbio.1002171)
Supplement: S3 Table — (DOCX) [file pbio.1002171.s003.docx]

Experimental Evidence for Phonemic Contrasts in a Nonhuman Vocal System: Engesser et al

**Supplementary Table**

S3 Table. Mahalanobis D-squared distances of among element comparisons in DFA and associated probability values in parentheses based on t distributions with *DF*=*N*-2.

| Element | F1 | F2 | P1 | P2 | P3 |
| --- | --- | --- | --- | --- | --- |
| Flight 1 (F1) | - |  |  |  |  |
| Flight 2 (F2) | 18.4 (**<0.001**) | - | - | - |  |
| Prompt 1 (P1) | 17.4 (**<0.001**) | 0.7 (0.4) | - | - |  |
| Prompt 2 (P2) | 1.4 (0.2) | 22.9 (**<0.001**) | 20.1 (**<0.001**) | - |  |
| Prompt 3 (P3) | 14.1 (<**0.001**) | 1.4 (0.2) | 0.2 (0.8) | 16.1 (**<0.001**) | - |
