# Supplementary material for: Experimental Evidence for Phonemic Contrasts in a Nonhuman Vocal System
Source: PLoS Biol. 2015 Jun 29;13(6):e1002171. doi: 10.1371/journal.pbio.1002171 (PMC4488142; doi:10.1371/journal.pbio.1002171)
Supplement: S1 Text — (DOCX) [file pbio.1002171.s004.docx]

Experimental Evidence for Phonemic Contrasts in a Nonhuman Vocal System: Engesser et al

***Supplementary Methods 1: Analysis of call-element parameters***

We first determined whether the five extracted acoustic parameters captured significant variation among the five call elements (F1, F2, P1, P2, P3) using a multivariate analysis of variance (MANOVA). To this end, we fitted the five call parameters as response terms, the 5-level factor pertaining to the five elements as the fixed effect, and the individual to which the elements belonged as a blocking function. Overall, there was a highly significant effect of element type on the response terms considered, indicating that our parameters captured meaningful variation in element structure (*F*_20,124_=9.57, *P*<0.001; *Wilks λ*=0.045). Next, we identified those element parameters that independently captured variation in call elements using univariate ANOVAs, in which any correlated element parameters (S1 Table) were fitted as a co-variate. These analyses revealed that three call parameters tended to vary independently among the five elements: frequency range (*F*_4,40_=25.05, *P*<0.001 controlling for element duration), start frequency (*F*_4,40_=2.58, *P*=0.058 controlling for end frequency) and end frequency (*F*_4,40_=3.38, *P*=0.018 controlling for start frequency). Element identity failed to account for significant variation in time to peak frequency (*F*_4,41_=0.12, *P*=0.98) or element duration after controlling for frequency range (*F*_4,40_=1.44, *P*=0.24).

Fitting the 3 significant (or near significant) acoustic parameters into a Discriminant Function Analysis (DFA) revealed that Discriminant Function 1 (DF1) explained 95% of the variance (*χ^2^*=145.56, *DF*=12, *P*<0.001) and Discriminant Function 2 (DF2) explained the remaining 5% (*χ^2^*=17.27, *DF*=6, *P*=0.008). Consideration of the eigenvalues and standardised vector loadings suggested that DF1 is heavily weighted to frequency range compared with the other two traits, while DF2 is positively weighted to start and end frequency and negatively to frequency range; thus DF2 explains the contrast between frequency range and start/end frequency (S2 Table). Fig. 1B shows the dominant effect of function 1 over function 2 on element discrimination (i.e. greater discrimination along the x than y axis), suggesting a primary effect of frequency range on element discrimination.
