# Supplementary material for: Experimental Evidence for Phonemic Contrasts in a Nonhuman Vocal System
Source: PLoS Biol. 2015 Jun 29;13(6):e1002171. doi: 10.1371/journal.pbio.1002171 (PMC4488142; doi:10.1371/journal.pbio.1002171)
Supplement: S2 Text — (DOCX) [file pbio.1002171.s005.docx]

Experimental Evidence for Phonemic Contrasts in a Nonhuman Vocal System: Engesser et al

***Supplementary Methods 2: Bird locations during aviary playbacks***

If one individual was tested, playbacks were broadcasted using a XMI X-mini II speaker placed in the empty aviary compartment 2 next to the compartment 3 of the focal bird. If two individuals were tested, the focal individuals were kept in two different compartments with the speaker in an empty compartment in-between (i.e. bird A in compartment 1; speaker in compartment 2 and bird B in compartment 3). When a third individual was tested, the two first birds were distributed in compartments as described for two birds; the third was kept in the compartment furthest away (i.e. compartment 6). In this case, playbacks were broadcasted with a Sony SRS-A27 stereo speaker system with one speaker again in the empty compartment in-between the first two test subjects (compartment 2), and the second in the empty compartment next to the third individual (compartment 5).
